# Editorial Extracts and Gleanings

**Published:** 1874-02

**Authors:** Swann M. Burnett

**Affiliations:** Knoxville, Tenn.


### EXTRACTS AND GLEANINGS

BY SWAN M. BURNETT, M.D., KNOXVILLE, TENN.

*Treatment of Sore Nipples.*—Few of the diseases attendant upon the purturient state are more annoying to the physician than sore nipples. The following remarks on the subject, from Prof. Barker, of Bellevue Hospital (*New York Medical Record,*) will be of material assistance to the perplexed practitioner who wants to know "what to do."

"If the nipple is inflammed apply a poultice until the inflammation is subdued, and then apply a solution of nitrate of lead in glycerine, ten grains to the ounce. This is also the most complete and perfect prophylactic against the occurrence of sore nipples that I know of. This solution should be applied immediately after nursing, having first washed the nipple perfectly clean. It may be used even stronger, 15 grs. or even ʒi., but as a rule 10 grs. is sufficient.

If there is an abrasion or raw surface it must be protected. For this, nothing has succeeded better with him than comp. tinct. benzoin. Wipe the nipple dry after the child has nursed, and apply with a camel's hair brush four or five coats of the tinct. When the fissure is at the base of the nipple, very small it may be, but accompanied by most severe and agonizing pain, touch the fissure with a fine point of nitrate of silver, and apply over it the tinct. of benzoin as before. If the inflammation and ulceration have destroyed the surface of the nipple, remove the child from the breast and use a breast pump, or empty the breast by rubbing.

He then uses: ℞ Rose ointment, ℥i: carb. magnesia, ℈i: calomel, gr. xxx. M. Rub together carefully, and it is better to have it prepared every twentyfour or thirty-six hours. If the child nurses at all, it must be through an artificial shield, the best of which is made from the cows teat. If these are not precurable, use one with a broad base, known as the L shaped glass. The ordinary ones are simply abominable.

*Exudations of Diphtheria and Croup.*—These, says Dr. Jabez Hogg, of London, a high authority on such subjects, have a sharply defined histological difference. The diphtheritic exudation is a dense, compact, opaque, felt like membrane, firmly adherent and not removable spontaneously, and when detached by force comes away in fragments, leaving a broken, bleeding surface. Under a × 350 power, it consists of fibrous and connective tissue, shrunken and compressed cells (epithelial, muscular, glandular and even cartilaginous) fat molecular, muco-purulent or glandular corpuscles, crystals, starch granules fungus spores, and other foreign bodies. The croupous cast, is, on the other hand, a delicate semi-transparent, often gelatinous exudation, not so intimately connected with the subjacent mucous membrane, but that it is easily seperable as an imperfect cast, which is often thrown off during a fit of coughing. Under the same magnifying power, it shows pavement and ciliated columnan epithelium and a homogeneous transparent elbuminous substance, (never truly fibrous) entangling detached apithelial cells or their contents, fat, mucous corpuscles, and a few foreign bodies: rarely do they show fungus spores. They continue probably to be thrown off soon after their formation, and appear to partake of the nature of an excessive cell proliferation rather than a true exudation; they are essentially an epithelial layer cast off, resembling the skin shed by some of the lower animals. There appears then to be something more than a mere "clinical tradition" that separates the two diseases. We believe it is a generally accepted fact among the more advanced scientific practitioners that, diphtheria is a constitutional disease, while true croup is more localized in its character. These investigations certainly points that way, and is another instance of corroborative evidence furnished by the microscope in medical questions under discussion.

*An Anti-Miasmatic Tree.*—*The London Daily Telegraph* has published the following account of this important subject: At the meeting of the French Academy of Science, a very interesting paper was read by Mr. Ginibert, on the alledged febrifugal properties of the *Eucalyptus Globulus* which is said to have the curious and valuable power of destroying the malarious element in any atmosphere where it grows. It belongs to a family of trees indigenous to New South Wales, which the colonists call gum-tree. The tree absorbs an deal immense of water from the earth, and at the same time emits an aromatic odor, which has perhaps, something to do with the beneficial influence attributed to it. Where it is thickly planted in marshy districts, the subsoil is said to be drained in a little while by the excessive piping. At a farm twenty miles from Algier, situated on a river, and noted for its extremely pestilential air, about 13,000 eucalpyti were planted. In the same year, at the time when the fever season used to set in, not a single case occurred; yet the trees were not more than nine feet high. Since then complete immunity from fever has been maintained. Throughout Cuba, at points where the tree has been introduced marsh diseases are fast disappearing. I have a botanical friend to whom I showed this extract, who says the tree can be made to grow on our Southern coast, and as its introduction and cultivation seems to be attended with little or no difficulty, the experiment of transplanting it to some of our marshy districts should be, by all means attempted. The sunflower is said to possess similar properties. In this connection the *Science Gossip* says: "There are more wonders in the vegetable world than are dreamed of in our philosophy. How passing strange for example, that property of the the papaw tree to turn meat tender! A joint of mutton, steeped in a solution of its juice, becomes instantly succulent, and the flesh of animals fed on its leaves "melts in the mouth," upon cooking."

*Another Test for Morphia.*—Mr. T. B. Graves presented the follow ingtest for morphia to the last meeting of the British Plarmaceutcal Association, which is said to be very delicate. The alkaloid must first be separated from other substances—then heat the suspected substance with about six drops of pure sulphuric acid, and then add a very small quantity of pure perchlorate of potassium. If morphia is presented, the liquid immediately surrounding the perchlorate will at once assume a deep brown color, which will soon extend over the greater part of the acid. Warming increases the delicacy of the test. It is essential that the perchlorate shall be free from the chlorate.—*Boston Journal of Chemistry.*

*New Test for Albumun.*—This is picric acid, and is recommended by M. Gallipe, as very sensitive. Picric acid causes no precepitation in normal urine, but if a drachm or two of a solution of it be put in a test tube and a few drops of urine be allowed to fall into it, if any albumen is present, it will show itself by a characteristic white line through the testing solution.

*Glanular Lids* are treated successfully in Rossevelt Hospital, New York, by liq: plum: aubacet: with dropping two drops of it undiluted in the eye every three or four days.

*Adulterations of Tea.*—One of the most common of those is the leaves of the sloe. (*Pruners Spinosa.*) The *Science Gossip* says: The sloe leaf is serrated from the stem to the apex, while the serrations of the tea leaf begin one-quarter to one-half inch from the stem. The serations of the sloe extend to the edge of the leaf—while those of the tea do not extend quite to the edge, and besides that, they recurve. The tip of the sloe leaf is acute, in the tea emarginate. Stomota of sloe semilunar is shape of tea reniform. Other adulterations are the leaves of the strawberry or sage and willow herb, (*epilobium angustifolium.*)

*How to give Phosphorus.*—We often want to administer phosphorus, and many physicians think they can do it by giving phosphoric acid. That, however, is a mistake, you can obtain the effect of phosphorus only by administering phosphorus, and for doing that, the *Boston Journal of Chemistry* gives the following formula—dissolve a grain of phosphorus in six drams of chloroform and add two ounces of good glycerine, and shake well. This makes a permanent solution, keeps well, and is pleasant. Dose, a teaspoonful three times a day.

*Liquid Nourishment for Sick Stomach.*—An egg well beaten up, to which add one piut of good milk and one pint of cold water and salt to make it palatable; let it be boiled, and when cold any quantity of it may be taken. If it curds it is useless.—*Ex.*

*Spermatorrhoea.*—In the Lamer Hospital the following is found more than ordinarily efficient. ℞ Feri: sulph:, acid nit:, *aa*, ℥i; aquæ ℥i; quin: sulph: ℥ss.; acid arsen: et strych: sulph, *aa.* gr. i—M. S. twenty drops three times a day.

---

Diabetic Bread.—In a somewhat recent number of the *Bulletin Géněral de Thěrapeutique*, M. Dannecy proposes the use of bread made from roasted flour for diabetic patients, instead of gluten biscuit. He asserts that roasted starch cannot be converted into glucose, and that bread made out of the various farinas so torrefied is greedily eaten by patients who have been restricted to the ordinary preparation of gluten until they have become thoroughly disgusted. Moreover, under its use the thirst lessens, and the digestive arrangements are markedly ameliorated.

Adhesive Plaster.—According to Otto Facilides, adhesive plaster, which has become brittle by age, and has lost its adhesive qualities, may be rendered adhesive again by coating it with oil of turpentine, by means of a sponge, and leaving it exposed for a day.

